# Investigating animal reservoirs for hepatitis E virus in Bangui, Central African Republic

**DOI:** 10.1371/journal.pone.0300608

**Published:** 2024-03-15

**Authors:** Marina Prisca de Marguerite Nombot-Yazenguet, Abdou Fatawou Modiyinji, Vianney Tricou, Alexandre Manirakiza, Richard Njouom, Narcisse Patrice Joseph Komas

**Affiliations:** 1 Viral Hepatitis Laboratory, Institut Pasteur de Bangui, Bangui, Central African Republic; 2 Department of Virology, Centre Pasteur of Cameroon, Yaoundé, Cameroon; 3 Epidemiological Service, Institut Pasteur de Bangui, Bangui, Central African Republic; University of Ghana, GHANA

## Abstract

**Background:**

Hepatitis E virus (HEV) is a major cause of enterotropic viral hepatitis, a major public health problem in many developing countries. In Central African Republic (CAR), HEV genotypes 1, 2, and 3 have been found to have an impact on human health. However, data on HEV in animal reservoirs are still lacking for CAR. Here, we investigated the presence of HEV in farmed pigs and goats in Bangui, the capital city of CAR, using molecular methods.

**Methodology:**

In a prospective study, fecal samples from 61 pigs and 39 goats from farms in five districts (2^nd^, 4^th^, 6^th^, 7^th^, 8^th^) of Bangui were collected and tested for HEV RNA by real-time RT-PCR. The samples were further analyzed by nested-PCR and sequenced to determine the genotype and subtype to which the virus belong.

**Results:**

In total, 22/100 (22.0%) feces samples were successfully amplified for HEV RNA by real time RT-PCR. All positive samples were from pigs (22/61; 36.1%), while all goat samples were negative (0/39). Twelve HEV RNA samples (12/22 or 54.5%) were successfully amplified by nested RT-PCR, and subsequently sequenced. Phylogenetic analysis revealed that the obtained sequences clustered with subtype 3h and were genetically related to the human HEV sequences from CAR.

**Conclusion:**

This study confirms that pigs constitute an HEV reservoir, with genotype 3 being the major circulating strain. Further studies are needed to investigate other local reservoirs and to improve knowledge of the molecular epidemiology of HEV in CAR.

## Introduction

Hepatitis E virus (HEV) is a major cause of enterotropic viral hepatitis, which has become a major public health problem in many developing countries in Asia and Africa [1]. According to the World Health Organization (WHO), approximately 20 million HEV infections occur annually worldwide, resulting in 3.3 million symptomatic cases and approximately 44,000 deaths [2]. The viral genome is a single-stranded positive-sense RNA of approximately 7,200 nucleotides with three open reading frames (ORF1-3). ORF1 encodes the non-structural proteins for genome replication, ORF2 encodes the capsid protein, and ORF3 encodes a small multifunctional protein [3–5]. Rat HEV and ferret HEV contain an additional open reading frame (ORF4) of still unknown function [6]. HEV belongs to the family *Hepeviridae* and has been assigned to the subfamily *Orthohepevirinae* for strains infecting mammals and birds and to the subfamily *Parahepevirinae* for strains infecting fish [7]. The subfamily *Orthohepevirinae* is divided into four genera, including the genera *Paslahepevirus* and *Rocahepevirus*, which infect humans, domestic and wild mammals, the genus *Chirohepevirus*, which infects bats, and the genus *Avihepevirus*, which infects birds. Members of the species *Paslahepevirus balayani* contain eight HEV genotypes (HEV-1 to HEV-8), of which five genotypes, HEV-1, HEV-2, HEV-3, HEV-4, and HEV-7 are responsible for human infections [7]. HEV-1 and 2 are restricted to humans and are transmitted feco-orally. They have been responsible for outbreaks in low socio-economic settings, particularly in Africa and Asia [8]. HEV-3 and 4 are zoonotic and infect humans and several other species such as pigs [9]. These two genotypes are the major cause of sporadic human infection in developed countries and are transmitted to humans through the consumption of infected meat [10]. HEV-5 and 6 infect wild boar; HEV-7 and 8 infect dromedary and Bactrian camels, respectively [11]. HEV genotypes 1, 2, 3 and 4 were subdivided into subtypes by phylogenetic analysis: 7 for genotype 1 (1a-1g), 2 for genotype 2 (2a and 2b), 13 for genotype 3 (3a-3m) and 9 for genotype 4 (4a-4i) [12]. In the Central African Republic (CAR), several studies have been carried out on HEV, mainly in human populations, and HEV-1e and HEV-2 were the main genotypes identified [13, 14]. More recently, HEV-3h was detected in the serum sample of a child suffering from HIV infection and severe acute malnutrition (the strain was initially identified as belonging to genotype 3c [15] due to high homology with strains circulating in France, but was later reassessed as genotype 3h following the subsequent revisions of the HEV phylogeny [12, 16, 17]). There are few studies on the molecular epidemiology of HEV in potential animal reservoirs in CAR. To the best of our knowledge, this study provides the first report on the genome characterization of HEV in two domestic animals species (pigs and goats), which are the most widely reared in the city of Bangui.

## Material and methods

### Study site and sample collection

This study was conducted from January to October 2021 in Bangui, the capital city of CAR. Bangui covers an area of 67 Km^2^, with an estimated population of 1,425,276 in habitants according to the “Institut Centrafricain des Statistiques et des Etudes Economiques et Sociales” (ICASEES) [18]. Bangui is administratively divided into eight arrondissements, 16 groups and 205 neighbourhoods. An initial survey was conducted to identify the farms. Subsequently, the domestic animal keepers were given verbal and written information about the study and gave their written consent for their animals to be sampled. Fecal samples were then collected from domestic pigs and goats in five districts (2^nd^, 4^th^, 6^th^, 7^th^, 8^th^) of Bangui. The day before sampling, the farmer was notified to bring the animals (pigs and goats) together in their respective pens. On the day of sampling, a team consisting of three (3) veterinarians and a biologist collected the animals’ samples while respecting hygienic conditions. Prior to sampling, the animal was clinically examined, and the anus was disinfected with alcohol. Fecal samples were collected by inserting a swab into the rectum of the recumbent animal and placed in sterile tubes. All samples were labelled and transported at 4˚C to the Institut Pasteur de Bangui (IPB) and then stored at -80°C. They were then sent to the Centre Pasteur of Cameroon (CPC) for molecular testing. In addition, information such as age, sex and area of collection was recorded on a sheet for each domestic animal collected. This study was approved by the Institutional Ethics Committee of the Faculty of Health Sciences of the University of Bangui (16/UB/FACSS/CSCVPER/11) and authorized by Ministry of Livestock of the Central African Republic under the number 009/MESA/DIRCAB/CMSA.21.

### Molecular analysis of HEV

Fecal samples were suspended in 10% (w/vol) phosphate-buffered saline (PBS; pH 7.2) and clarified by centrifugation at 12,000 g for 10 min at 4°C. RNA was extracted from 140 μl of fecal supernatant using the QIAamp Viral RNA mini Kit (Qiagen Courtaboeuf, France) according to the manufacturer’s instructions.

Two molecular amplification techniques, namely real-time RT-PCR and nested RT-PCR were used. Real-time RT-PCR is a very sensitive method with results obtained in a short time (between 30 min to 2 hours maximum) but it did not allow us to obtain the sequences of the amplicons. For this reason, we subsequently used a nested RT-PCR which allowed us to proceed to amplicon sequencing and phylogenetic analysis of circulating HEV strains.

Detection of HEV RNA was performed using the CFX Connect Real-Time PCR System (Bio-Rad, Hercules, California) according to a previously described protocol [19]. The QIAgen One step RT-PCR kit (QIAgen) was used for the amplification reaction. The primers used (20 μM) were the sense primer HEV5260 (5´-GGTGGTTTCTGGGGTGAC-3´) and the antisense primer HEV5330 (5´-AGGGGTTGGTTGGATGAA-3´). The probe used was HEV5283 (15 μM; 5´-FAM-TGATTCTCAGCCC TTCGC-TAMRA-3´). The following thermal profile was used: 50°C for 30 min, 95°C for 15 min, followed by 45 cycles of 94°C for 1 min, 51°C for 1 min and 72°C for 1 min. The manipulation was validated when the positive control showed a sigmoidal curve with Ct <37 while the negative control showed no curve. Samples positive by real-time RT-PCR were selected for amplification of a portion of ORF2 by nested RT-PCR using Perkin Elmer Gene Amp PCR System 9700 according to a previously described protocol [20]. The first step of our nested RT-PCR was performed using a SuperScript™ III One-Step RT-PCR System with Platinum Taq (Life Technologies Corporation, USA) in a final volume of 50 μL containing 0.2 μM of each primer (sense primer HEV-5920S: 5´-CAAGGHTGGCGYTCKGTTGAGAC -3´ and anti-sense primer HEV-6425A: 5´- CAAGGHTGGCGYTCKGTTGAGAC -3´), 2.3 mM MgSO4, 200 μM dNTPs and 10 μL of RNA extract. The thermal profile was as follows: 50°C for 30 min, 94°C for 2 min, followed by 40 cycles at 94°C for 15 sec, 60°C for 30 sec and 72°C for 1 min and 72°C for 5 min. For the second step, 5 μL of the products from the first step were used in a final volume of 50 μL containing 1.25 U of Taq polymerase (Life Technologies Corporation, USA), 1.5 mM MgCl2 and 0.2 μM of each primer (sense primer HEV-5930S: 5´- GYTCKGTTGAGACCWCBGGBGT -3´ and anti-sense primer HEV-6334A: 5´- TTMACWGTRGCTCGCCATTGGC -3´). The thermal profile consisted of 94°C for 5 min, 40 cycles of 94°C for 30 sec, 55°C for 30 sec, 72°C for 1 min 30 sec and 72°C for 10 min. The manipulation was validated when, after electrophoresis on an agarose gel, the positive control showed a band of the expected size, whereas the negative control did not show any band. The second amplification product of 467 bp was sequenced using the BigDye Terminator Cycle Sequencing on an ABI PRISM 3100 Genetic Analyzer (Applied Biosystems, Foster City, CA, USA). Genotyping was performed by phylogenetic analysis. Twelve consensus sequences were obtained using CLC Main Workbench 5.5.2. The phylogenetic tree was constructed using MEGA 6.0 software by maximum likelihood (ML) method with references sequences for the different genotypes and subtypes as described [12]. The confidence level of the ML tree was assessed by bootstrapping with 1000 replicates.

### Statistical analysis

Prevalences are expressed as percentages; Fisher’s exact test was used to analyze the association between HEV RNA detection and various demographic parameters. The significance level was P < 0.05. All analyses were performed using Stata statistical software (Stata- Corp LP, College Station, TX, USA).

## Results

### Characteristics of collected samples

A total of 100 samples were collected from pigs (n = 61) and goats (n = 39). For both animals, the age ranged from less than 6 months to more than 6 months, with more samples from females (58/100; 58.0%) (Table 1).

**Table 1:** Characteristics of samples collected in the study.

Sociodemographic characteristics	Total N = 100	Pig n = 61 (61.0%)	Goat n = 39 (39.0%)
**Sex of animal**			
Female	58	33 (56.9)	25 (43.1)
Male	42	28 (66.6)	14 (33.3)
**Age group (months)**			
≤ 6	71	52 (73.2)	19 (26.8)
> 6	29	9 (31)	20 (69)
**District (arrondissement)**			
2^nd^	14	14 (100)	0 (0)
4^th^	51	35 (68.6)	16 (31.3)
6^th^	20	0 (0)	20 (100)
7^th^	10	10 (100)	0 (0)
8^th^	5	2 (40)	3 (60)

**Fig 1:**
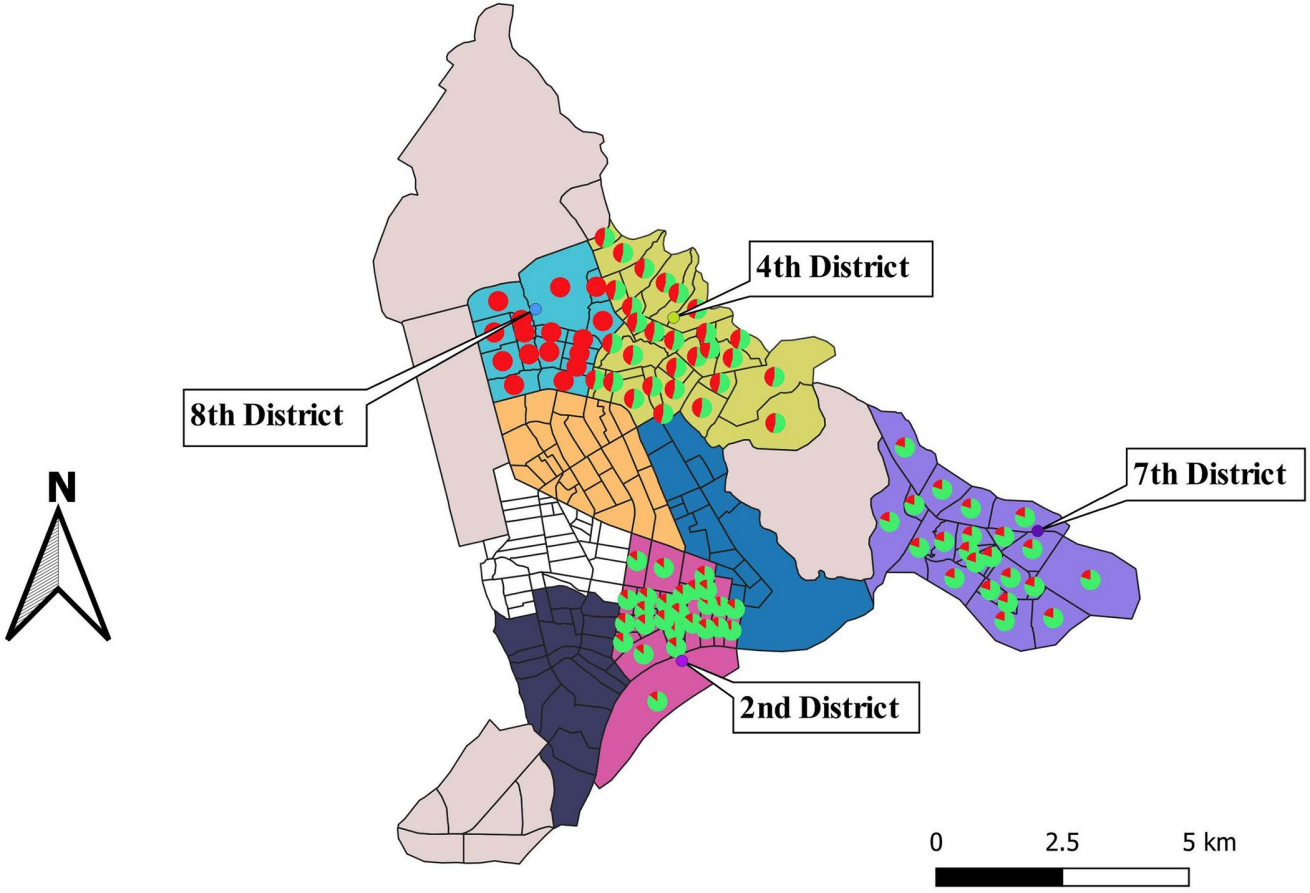
Map of Bangui showing the districts and farms where fecal samples were collected from domestic pigs and goats. The pie chart shows the proportions of HEV RNA positive (red) and negative (green) samples. This map was created with QGIS software, https://www.qgis.org/fr/site/ from the Bangui shapefile downloaded from https://data.humdata.org/dataset/cod-ab-caf).

**Fig 2:**
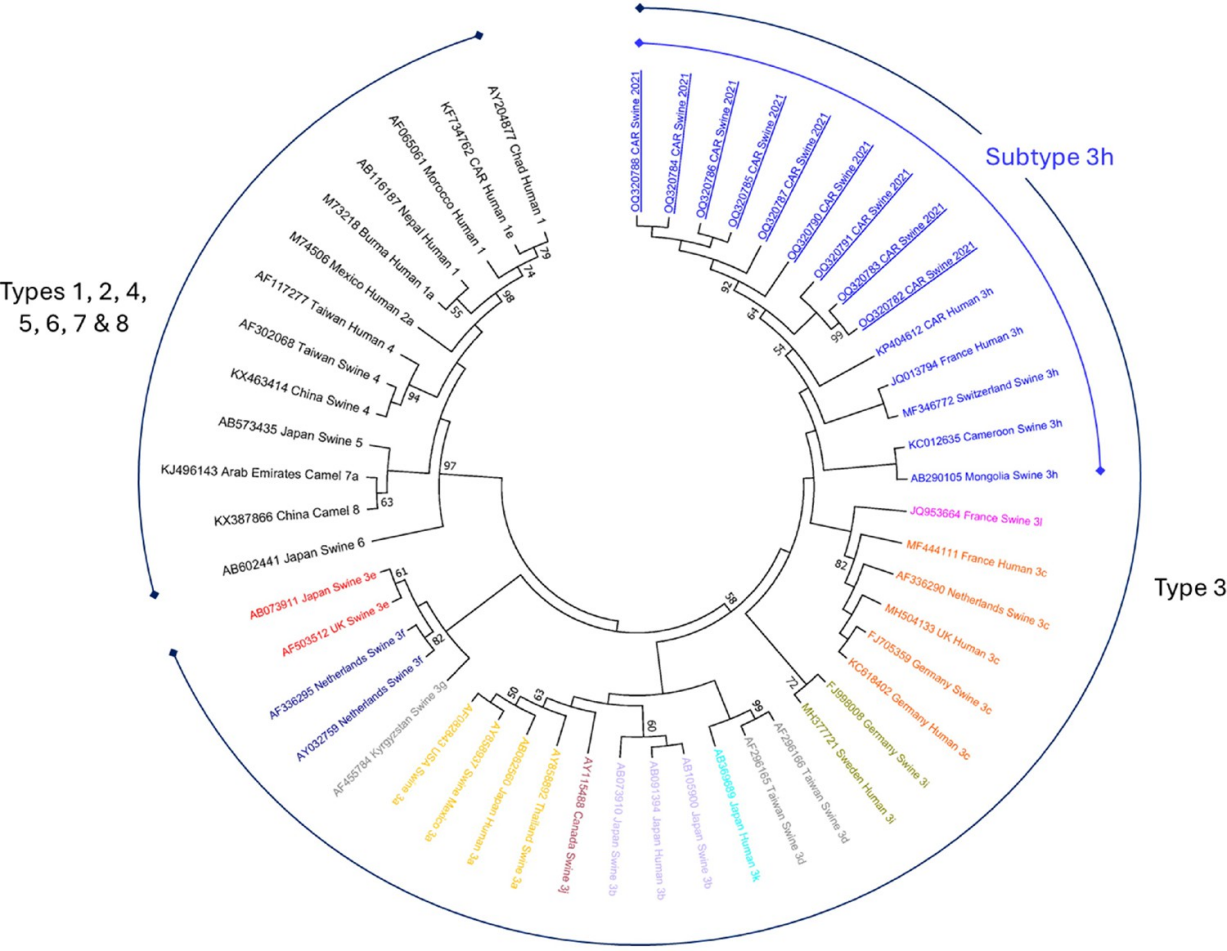
Phylogenetic tree constructed with mega 6.0 program using the maximum likelihood method. The bootstrap consensus tree inferred from 1000 replicates is taken to represent the evolutionary history of the taxa analyzed. Numbers in the tree represent the bootstrap value (bootstrap values below 50% are not shown at the nodes). The analysis involved 51 sequences. The sequences identified in this study are underlined.

HEV RNA was detected by real-time RT-PCR in 22 (22.0%) of 100 fecal samples tested from 61 pigs and 39 goats. All 22 amplified HEV RNA samples were found in pigs (22/61, 36.1%), while all goat fecal samples were negative (0/39, 0.0%). The 22 fecal samples with amplified HEV RNA by real-time RT-PCR were selected for nested RT-PCR analysis. Of these, 12 out of 22 samples were amplified (54.5%) and successfully sequenced. As shown in Table 2, the amplified HEV RNA was similar in female and male pigs. With regard to the amplification rate by age group, the RNA detection rate did not differ between the age groups less than or equal to 6 months of age and more than 6 months of age (Fisher’s exact test; *P*  =  0.8). However, the association between HEV RNA detection and demographic characteristics of the domestic animals was not statistically significant. Samples were collected from domestic pig and goat farms in five districts. Four of these farms were found to have amplified pig HEV RNA. A total of 16/35 (45.7%) of the amplified HEV RNA samples were found in the 4^th^ district (Fig 1). Sequencing and phylogenetic analysis showed that all porcine HEV RNA sequences belonged to genotype 3, and clustered within subtype 3h (Fig 2). Twelve sequences obtained in this study are available in GenBank under accession numbers OQ320780-OQ320791.

**Table 2:** Detection of HEV RNA in pig fecal sample by real time RT-PCR.

Demographic variables	Number of specimens	HEV RNA Positive N (%)	*P*
**Sex of animal**			0.9
Female	33	12 (36.4)	
Male	28	10 (35.7)	
**Age group (months)**			0.8
≤ 6	52	19 (36.5)	
> 6	9	3 (33.3)	
**District (arrondissement)**			0.03
2^nd^	14	2 (14. 3)	
4^th^	35	16 (45.7)	
7^th^	10	2 (20)	
8^th^	2	2 (100)	

## Discussion

This study investigates for the first time the presence of HEV in domestic animals identified as possible reservoirs of HEV in several districts of Bangui, the capital city of CAR. In this study, HEV RNA was found in pig feces but not in goat feces. These results confirm that pigs are an important reservoir of HEV [20]. This finding is consistent with a report from Ghana where HEV RNA was not detected in goats [21]. However, detection of HEV RNA in goats has been reported in other studies from China and Italy, where HEV RNA was detected in 74.07% and 9.2% of goat fecal samples, respectively [22, 23]. Our finding could be explained by the fact that all the goats tested were less exposed, or that HEV infection is rare in this species, or that the diagnostic methods used in our study were not fully appropriate for this species [24]. The overall prevalence of fecal HEV RNA in the pig population was 22/61 (36.1%) which is higher than reports from Ethiopia (12%) [25], Cameroon (5.9%) [20] and Congo (2.5%) [26]. We observed no clear difference between the presence of HEV and the sex and age group of the pig, similar to reports from Cameroon and Madagascar [20, 27]. Our results are not in agreement with previous studies where young pig were much more likely to have HEV contamination [28–32]. It has been suggested that contamination of these young pigs may occur during the suckling period from virus-shedding sows [26, 30]. Although domestic pigs and goats from five districts were included in this study, HEV was only detected in pigs from four of the districts (2^nd^, 4^th^, 7^th^ and 8^th^). Pigs from farms located in the 4^th^ district had the highest frequency of positive samples. This district is a densely populated area with a high density of pig farming and production compared to other districts. It is also the district where most pig samples were collected. In addition, it is characterized by poor hygienic conditions with very little space for rearing, which may allow cross-contamination between animals and allow HEV to spread. This may explain the high detection rate of HEV observed in this district. On the other hand, there are very few livestock farms in the 8^th^ arrondissement, which borders the 4^th^ arrondissement, where it was very difficult to collect suitable samples. The farming systems practiced include traditional free-range farming [33], where pigs are housed or allowed to roam freely during the day in search of food and water and are locked up at night. They visit waste piles and stagnant bodies of water, contaminating these areas with feces and urine. As reported in a study in Nigeria [29], these husbandry systems predispose pigs to various infections, including HEV, and thus facilitating onward transmission to humans, especially in environments where there is a close association between pigs and humans. The presence of HEV RNA in certain domestic pig farms, particularly in the 4^th^ arrondissement of Bangui, may be the source of a jump in species contamination between pigs and humans. The human population living in this area must therefore be informed about HEV prevention.

Similar to other reports in Africa, HEV genotype 3 was identified in porcine fecal samples [20, 26, 34]. However, more pig and human HEV sequences are needed to clarify the origin of HEV strains in the pig population and the possibility of pig-to-human transmission of HEV. The proximity of our sequences to those from other countries raises the question of the origins of the pigs analysed, but lack of information prevents us from investigating this further. Future studies will be necessary to answer some of the unanswered questions about the epidemiology of HEV in CAR.

Finally, this study should also be complemented by a serological study of HEV in animals in CAR. This is a limitation of our study, which we plan to address in a more comprehensive study in several CAR cities.

## Conclusion

This study provides the first evidence that pig populations are a reservoir for HEV infection in CAR. The results showed the prevalence of HEV-RNA in pig farms in several districts of Bangui and the circulation of genotype 3h in the pig population. Further studies are needed to investigate other reservoirs of HEV and to improve knowledge of the molecular epidemiology of HEV in CAR.

## Supporting information

S1 Table HEV database of study. (PDF)
