# Supplementary material for: Investigating animal reservoirs for hepatitis E virus in Bangui, Central African Republic
Source: PLoS One. 2024 Mar 15;19(3):e0300608. doi: 10.1371/journal.pone.0300608 (PMC10942039; doi:10.1371/journal.pone.0300608)
Supplement: S1 Table — (PDF) [file pone.0300608.s001.pdf]

Supplemental Table 1: HEV Database of study

Coded	Included date	Sampling date	District	Locality	Animal species	Age/ month	Sexe	Type of collection	Q-RT-PCR/Ct-value	Conclusion Q-RT-PCR	Conventional RT-PCR	Genotype
SP01100221	01/02/2021	10/02/2021	4th	Votongbo 1	Swine	4	F	Fecal	28,000	Positive	Positive	HEV-G3
SP02100221	01/02/2021	10/02/2021	4th	Votongbo 1	Swine	4	M	Fecal	28,100	Positive	Positive	HEV-G3
SP03100221	01/02/2021	10/02/2021	4th	Votongbo 1	Swine	4	M	Fecal	29,300	Positive	Positive	HEV-G3
SP04100221	01/02/2021	10/02/2021	4th	Votongbo 1	Swine	4	F	Fecal	24,600	Positive	Positive	HEV-G3
SP05100221	01/02/2021	10/02/2021	4th	Votongbo 1	Swine	2	F	Fecal	39,700	Positive	Negative	
SP06100221	01/02/2021	10/02/2021	4th	Votongbo 1	Swine	1	F	Fecal	indetermined	négatif	Negative	
SP07100221	01/02/2021	10/02/2021	4th	Votongbo 1	Swine	2	F	Fecal	36,600	Positive	Negative	
SP08100221	01/02/2021	10/02/2021	4th	Votongbo 1	Swine	2	M	Fecal	indetermined	Negative		
SP09100221	01/02/2021	10/02/2021	4th	Votongbo 1	Swine	2	M	Fecal	29,800	Positive	Positive	HEV-G3
SP10100221	01/02/2021	10/02/2021	4th	Votongbo 1	Swine	2	M	Fecal	21,400	Positive	Positive	HEV-G3
SP11100221	01/02/2021	10/02/2021	4th	Votongbo 1	Swine	2	M	Fecal	20,700	Positive	Positive	HEV-G3
SP12100221	01/02/2021	10/02/2021	4th	Votongbo 1	Swine	2	F	Fecal	29,100	Positive	Positive	HEV-G3
SP13100221	01/02/2021	10/02/2021	4th	Votongbo 1	Swine	2	F	Fecal	27,900	Positive	Positive	HEV-G3
SP14100221	01/02/2021	10/02/2021	4th	Votongbo 1	Swine	8	M	Fecal	23,000	Positive	Positive	HEV-G3
SP15100221	01/02/2021	10/02/2021	4th	Votongbo 1	Swine	1	M	Fecal	indetermined	Negative		
SP16100221	01/02/2021	10/02/2021	4th	Votongbo 1	Swine	2	M	Fecal	indetermined	Negative		
SP17100221	01/02/2021	10/02/2021	4th	Votongbo 1	Swine	4	M	Fecal	indetermined	Negative		
SP18100221	01/02/2021	10/02/2021	4th	Votongbo 1	Swine	2	F	Fecal	indetermined	Negative		
SP19100221	01/02/2021	10/02/2021	4th	Votongbo 1	Swine	2	F	Fecal	indetermined	Negative		
SC20100221	01/02/2021	10/02/2021	4th	Votongbo 1	Goat	9	M	Fecal	indetermined	Negative		
SC21100221	01/02/2021	10/02/2021	4th	Votongbo 1	Goat	9	M	Fecal	indetermined	Negative		
SC22100221	01/02/2021	10/02/2021	4th	Votongbo 1	Goat	12	F	Fecal	indetermined	Negative		
SP23100221	01/02/2021	10/02/2021	4th	Votongbo 1	Swine	6	F	Fecal	indetermined	Negative		
SP24100221	01/02/2021	10/02/2021	4th	Votongbo 1	Swine	6	F	Fecal	indetermined	Negative		
SP25100221	01/02/2021	10/02/2021	4th	Votongbo 1	Swine	6	F	Fecal	24,400	Positive	Positive	HEV-G3
SP26100221	01/02/2021	10/02/2021	4th	Votongbo 1	Swine	6	F	Fecal	35,300	Positive	Negative	
SP27100221	01/02/2021	11/02/2021	4th	Votongbo 1	Swine	6	F	Fecal	indetermined	Negative		

SP28110221	01/02/2021	11/02/2021	4th	Votongbo 1	Swine	12	F	Fecal	indetermined	Negative		
SP29110221	01/02/2021	11/02/2021	4th	Votongbo 1	Swine	2	M	Fecal	indetermined	Negative		
SP30110221	01/02/2021	11/02/2021	4th	Votongbo 1	Swine	2	M	Fecal	36,300	Positive	Negative	
SC31110221	01/02/2021	11/02/2021	4th	Votongbo 1	Goat	12	F	Fecal	indetermined	Negative		
Sc31110221	02/02/2021	11/02/2021	4th	Londo 2	Goat	7	F	Fecal	indetermined	Negative		
SP32170221	02/02/2021	11/02/2021	4th	Londo 2	Goat	4	F	Fecal	indetermined	Negative		
SC33110221	02/02/2021	11/02/2021	4th	Londo 2	Goat	6	F	Fecal	indetermined	Negative		
SC35110221	02/02/2021	11/02/2021	4th	Londo 2	Goat	5	M	Fecal	indetermined	Negative		
SC36110221	02/02/2021	11/02/2021	4th	Londo 2	Goat	8	F	Fecal	indetermined	Negative		
SC37110221	02/02/2021	11/02/2021	4th	Londo 2	Goat	7	F	Fecal	indetermined	Negative		
SC38110221	02/02/2021	11/02/2021	4th	Londo 2	Goat	7	F	Fecal	indetermined	Negative		
SC39110221	02/02/2021	11/02/2021	4th	Londo 2	Goat	7	F	Fecal	indetermined	Negative		
SC41110221	02/02/2021	11/02/2021	4th	Londo 2	Goat	2	M	Fecal	indetermined	Negative		
SP42110221	02/02/2021	11/02/2021	4th	Londo 2	Goat	1	M	Fecal	indetermined	Negative		
SP43110221	02/02/2021	11/02/2021	4th	Ouham 1	Swine	7	M	Fecal	indetermined	Negative		
SP44110221	02/02/2021	11/02/2021	4th	Ouham 1	Swine	6	M	Fecal	35,300	Positive	Negative	
SP45110221	02/02/2021	11/02/2021	4th	Ouham 1	Swine	6	F	Fecal	indetermined	Negative		
SP46110221	02/02/2021	11/02/2021	4th	Ouham 1	Swine	6	F	Fecal	indetermined	Negative		
SP47110221	02/02/2021	11/02/2021	4th	Ouham 1	Swine	6	F	Fecal	indetermined	Negative		
SP48110221	02/02/2021	11/02/2021	4th	Ouham 1	Swine	7	F	Fecal	indetermined	Negative		
SP49110221	02/02/2021	11/02/2021	4th	Votongbo 1	Swine	6	F	Fecal	indetermined	Negative		
SP50110221	02/02/2021	11/02/2021	4th	Votongbo 1	Swine	4	M	Fecal	indetermined	Negative		
SC51150221	02/02/2021	15/02/2021	4th	Votongbo 1	Goat	5	M	Fecal	indetermined	Negative		
SC52150221	02/02/2021	15/02/2021	4th	Votongbo 1	Goat	4	M	Fecal	indetermined	Negative		
SC54150221	02/02/2021	15/02/2021	8th	Damala 2	Goat	12	F	Fecal	indetermined	Negative		
SC55150221	02/02/2021	15/02/2021	8th	Damala 2	Goat	4	M	Fecal	indetermined	Negative		
SC56150221	02/02/2021	15/02/2021	8th	Damala 2	Goat	4	F	Fecal	indetermined	Negative		
SP57150221	02/02/2021	15/02/2021	8th	Gongono 3	Swine	3	F	Fecal	27,300	Positive	Negative	
SP58150221	02/02/2021	15/02/2021	8th	Gongono 3	Swine	3	M	Fecal	23,500	Positive	Positive	HEV-G3
SC62160221	02/02/2021	16/02/2021	6th	Guitangola	Goat	12	F	Fecal	indetermined	Negative		
SC63160221	02/02/2021	16/02/2021	6th	Guitangola	Goat	12	F	Fecal	indetermined	Negative		
SC64160221	02/02/2021	16/02/2021	6th	Guitangola	Goat	12	F	Fecal	indetermined	Negative		

SC65160221	02/02/2021	16/02/2021	6th	Guitangola	Goat	4	M	Fecal	indetermined	Negative		
SC66160221	16/02/2021	16/02/2021	6th	Guitangola	Goat	4	M	Fecal	indetermined	Negative		
SC67160221	16/02/2021	16/02/2021	6th	Guitangola	Goat	7	F	Fecal	indetermined	Negative		
SC68160221	16/02/2021	16/02/2021	6th	Guitangola	Goat	12	F	Fecal	indetermined	Negative		
SC69160221	16/02/2021	16/02/2021	6th	Guitangola	Goat	12	F	Fecal	indetermined	Negative		
SC70160221	03/02/2021	16/02/2021	6th	Guitangola	Goat	24	F	Fecal	indetermined	Negative		
SC71160221	03/02/2021	16/02/2021	6th	Guitangola	Goat	5	M	Fecal	indetermined	Negative		
SC72160221	03/02/2021	16/02/2021	6th	Guitangola	Goat	1	F	Fecal	indetermined	Negative		
SC73160221	03/02/2021	16/02/2021	6th	Guitangola	Goat	24	F	Fecal	indetermined	Negative		
SC74160221	03/02/2021	16/02/2021	6th	92 logements	Goat	6	F	Fecal	indetermined	Negative		
SC75160221	03/02/2021	16/02/2021	6th	92 logements	Goat	6	M	Fecal	indetermined	Negative		
SC76160221	03/02/2021	16/02/2021	6th	92 logements	Goat	24	F	Fecal	indetermined	Negative		
SP77170221	03/02/2021	17/02/2021	7th	Mboko	Swine	7	M	Fecal	indetermined	Negative		
SP78170221	08/02/2021	17/02/2021	7th	Saint Paul 2	Swine	3	M	Fecal	indetermined	Negative		
SP79170221	08/02/2021	17/02/2021	7th	Saint Paul 2	Swine	3	M	Fecal	indetermined	Negative		
SP80170221	08/02/2021	17/02/2021	7th	Saint Paul 2	Swine	36	F	Fecal	indetermined	Negative		
SP81170221	08/02/2021	17/02/2021	7th	Saint Paul 2	Swine	3	F	Fecal	indetermined	Negative		
SP82170221	08/02/2021	17/02/2021	7th	Mboko	Swine	6	F	Fecal	indetermined	Negative		
SP83170221	08/02/2021	17/02/2021	7th	Mboko	Swine	6	M	Fecal	indetermined	Negative		
SP84170221	08/02/2021	17/02/2021	7th	Mboko	Swine	8	F	Fecal	indetermined	Negative		
SP85170221	08/02/2021	17/02/2021	7th	Mboko	Swine	6	F	Fecal	30,500	Positive	Negative	
SP86170221	09/02/2021	18/02/2021	7th	Mboko	Swine	7	F	Fecal	28,100	Positive	Negative	
SC87180221	09/02/2021	18/02/2021	6th	Guitangola	Goat	24	F	Fecal	indetermined	Negative		
SC88180221	09/02/2021	18/02/2021	6th	Guitangola	Goat	5	F	Fecal	indetermined	Negative		
SC89180221	09/02/2021	18/02/2021	6th	Guitangola	Goat	5	F	Fecal	indetermined	Negative		
SC90180221	09/02/2021	18/02/2021	6th	Guitangola	Goat	4	M	Fecal	indetermined	Negative		
SC91180221	09/02/2021	18/02/2021	6th	Guitangola	Goat	6	M	Fecal	indetermined	Negative		
SP91180221	09/02/2021	18/02/2021	2th	Kingoma	Swine	4	M	Fecal	indetermined	Negative		
SP92180221	09/02/2021	18/02/2021	2th	Kingoma	Swine	4	M	Fecal	indetermined	Negative		
SP93180221	09/02/2021	18/02/2021	2th	Kingoma	Swine	3	M	Fecal	indetermined	Negative		
SP94180221	09/02/2021	18/02/2021	2th	Kingoma	Swine	3	F	Fecal	indetermined	Negative		
SP95180221	09/02/2021	18/02/2021	2th	Kingoma	Swine	3	F	Fecal	indetermined	Negative		

SP96180221	09/02/2021	18/02/2021	2th	Kingoma	Swine	3	M	Fecal	indetermined	Negative		
SP97180221	09/02/2021	18/02/2021	2th	Kingoma	Swine	3	M	Fecal	indetermined	Negative		
SP98180221	09/02/2021	18/02/2021	2th	Kingoma	Swine	3	M	Fecal	indetermined	Negative		
SP99180221	09/02/2021	18/02/2021	2th	Kingoma	Swine	3	F	Fecal	indetermined	Negative		
SP100180221	09/02/2021	18/02/2021	2th	Kingoma	Swine	3	F	Fecal	indetermined	Negative		
SP101180221	09/02/2021	18/02/2021	2th	Kingoma	Swine	3	F	Fecal	indetermined	Negative		
SP102180221	09/02/2021	18/02/2021	2th	Kingoma	Swine	3	M	Fecal	33,200	Positive	Negative	
SP103180221	09/02/2021	18/02/2021	2th	Kingoma	Swine	4	M	Fecal	indetermined	Negative		
SP104180221	09/02/2021	18/02/2021	2th	Kingoma	Swine	9	F	Fecal	36,600	Positive	Negative	
